# Modulatory Effects of Plant Polyphenols on Bone Remodeling: A Prospective View From the Bench to Bedside

**DOI:** 10.3389/fendo.2019.00494

**Published:** 2019-07-23

**Authors:** Vanessa Nicolin, Nunziatina De Tommasi, Stefania Lucia Nori, Fulvia Costantinides, Federico Berton, Roberto Di Lenarda

**Affiliations:** ^1^Clinical Department of Medical, Surgical and Health Sciences, University of Trieste, Trieste, Italy; ^2^Pharmacy-DIFARMA Department, University of Salerno, Fisciano, Italy; ^3^School of Dental Sciences, University of Trieste, Trieste, Italy

**Keywords:** biomolecules, polyflavonoids, bone remodeling, titanium implant, coating surfaces

## Abstract

During the past, a more comprehensive knowledge of mechanisms implicated in bone resorption processes has driven researchers to develop a compound library of many small molecules that specifically interfere with the genesis of osteoclast precursors cells. Natural compounds that suppress osteoclast commitment may have therapeutic value in treating pathologies associated with bone resorption like osteoporosis, rheumatoid arthritis, bone metastasis, and periodontal disease. The present review is focused on the current knowledge on the polyphenols derived from plants that could be efficacious in suppressing osteoclast differentiation and bone resorption.

## Introduction

### Bone Cells and Cell Guest Outsider

The strength and integrity of the human skeleton depends on a delicate balance between bone resorption and formation. Bone resorption represents a final step due to the interaction between cells that involve resorption of bone (osteoclasts, OC), and that synthetize bone matrix (osteoblasts, OB). Bone remodeling allows to adapt bone tissue to mechanical forces and to maintain phosphocalcic homeostasis through coordinated steps of formation and resorption (1, 2).

This equilibrium is close controlled by physical parameters (i.e., mechanical stimulations) and several polypeptides (hormones, cytokines) (3, 4). In addition, bone remodeling cycle maintains the integrity of the skeleton through the balanced action of its cell types.

Cells responsible for bone resorption include (1) bone-forming osteoblast, a cell that produces the organic bone extracellular matrix; (2) the bone resorbing osteoclast that dissolves bone mineral and enzymatically degrades extracellular matrix and inorganic components (5); (3) osteocyte, the maturative phase of osteoblast that acts as a mechanosensor and like an endocrine cell (6); (4) the bone lining cell, that probably may have a role in coupling bone resorption to bone formation (7).

Basically, osteoblasts and osteoclasts (and their precursors) are the central players of bone remodeling and each factor that affects these cells, in the end, affects all the process.

Numerous signaling pathways carry on the activities of osteoblast and osteoclast cells and these include nuclear factor kappa-light-chain-enhancer of activated B cells (RANkL) and Bone Morphogenetic Protein (BMP) (8). RANkL/RANK signaling axes regulates osteoclast recruitment and sustenance in normal bone modeling and remodeling and is adversely regulated by osteoprotegerin (OPG) (9).

An asymmetry between bone resorption and formation can result in bone diseases for instance in osteoporosis and in other bone resorbing pathologies.

Osteoporosis appears to be related with an impairment of bone mass through the reduction of osteogenesis and an enhancement of osteoclastic bone resorption, which results in bone fractures (10). At the present treatment options are limited, having issues with their efficacy, and long-term use. For example, the antiresorptive drugs are efficacious in reducing fracture risk (11, 12). Anti-resorptive agents, such as bisphosphonates and denosumab decrease bone loss through the inhibition of the differentiation and catabolic activity of osteoclasts in part by promoting osteoclast apoptosis. Since bone formation is coupled to bone resorption, inhibition of bone resorption is followed by a decrease in osteoblast activity that affects to adequately restore bone mass and bone quality due to increased microdamage collagen formation (13).

By considering papers on adverse effects of pharmacotherapy (estrogens, bisphosphonates) in the treatment of osteoporosis, there is an increasing request for complementary and alternative medicine.

Plant based therapies (i.e., Polyphenols) are safe options at the common treatment of osteoporosis thanks to diminished side effects and costs; in this context various plant components have been assessed for their potential role in the management of osteoporosis. Polyphenols are phytochemicals normally found in the plant kingdom, whose several biological effects have been reported to be protective against chronic diseases, including neurodegenerative and cardiovascular pathologies, cancer, and bone resorption pathologies (14). Depending on the number of phenol rings they comprehend and on the radicals bound to them, polyphenols can be split into different groups: phenolic acids, flavonoids, stilbenes, tannins, coumarins, and lignans. Recent papers based on molecular characteristics of dietary polyphenols have outlined the advantage in their prevention and management of bone resorption diseases (15). Polyphenols can safeguard bone integrity through the decreasing of oxidative stress, the reduction of inflammation by proinflammatory signaling and the modulation of osteoblastogenesis/osteoclastogenesis, by an osteoimmunological action.

The aim of this review paper was achieved on the development of osteoclast-targeting plant polyphenols that could be a great value for the prevention or treatment of bone resorption.

## Methods

Studies for this narrative review were screened using the online database PubMed. Keywords used to search for articles included bone, polyphenols, iicarin, green tea polyphenols, anthocyanins, phloridzin, oleuropein, resveratrol, quercitrin, plorydzin, dried plum, citrus flavonoid, osteoblasts, osteoclasts, bone remodeling, bone resorption, periodontal disease, osteoporosis, oral health. Articles were selected based on the two aspects of the review (cellular and molecular behavior—clinical relevant scenarios). To discuss the cellular characteristics of the bone, milieu articles were selected based on how well they delineate the subject matter. Experimental studies dealing with the impact of polyphenols activity on bone cellular signaling were included. For the remaining section, were included studies based on the broadest spectrum of clinical applications presented and on the strength of evidence provided. Since the topic is relative newborn in its clinical utilization, most of the selected studies were *in vitro*.

## Polyphenol Compounds and Bone Metabolism

Polyphenols are natural molecules derived from plants isolated and characterized in the fruits, and vegetables. Bioactive phenolic compounds were known as health benefit components due to their antioxidant and anti-inflammatory features. Up to now, there are almost 8,000 recognized polyphenols of which ~500 are biologically active. They can be obtained from plentiful natural sources and even from food/ beverage industrial products with low costs, thus using resources in a feasible way (16). They can preserve bone health by the action of five possible mechanisms: (i) by decreasing of bone loss through the activity of antioxidants, (ii) by diminishing bone loss through anti-inflammatory action, (iii) by improving osteoblastogenesis, (iv) by reducing osteoclastogenesis, and (v) through osteoimmunological activity. Many flavonoids substances have displayed positive impact on bone metabolism. For example, dietary soy isoflavones suppress bone depletion in rodents and post-menopausic women (17). One of the most studied bioactive compounds during recent years is iicarin, a heavy flavonoid component of Chinese tea, which has been reported to have intriguing osteogenic properties both *in vitro* and *in vivo* (18–21).

**Figure 1:**
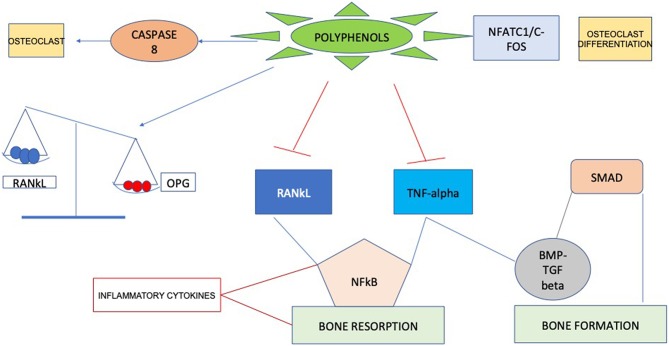
Schematic pathway illustrating the action of polyphenols on bone remodeling.

Recent researches have identified molecular targets in cell signaling pathways that influence bone structure. Some bioactive compounds seem to have bone anabolic action, which has important implications beyond the inhibition of bone resorption through suppressing osteoclast activation. Probably, the positive actions of these compounds are mainly due to their antioxidant characteristics, since they can act as scavengers of reactive oxygen species (ROS). Considering these properties polyphenols could affect bone metabolism through impairment of inflammatory mediators (22), such as cytokines, primarily involved in supporting osteoclast differentiation and resorption (23), consequently enabling to a decrease in bone resorption (Figure 1). Curcumin, for example, can enhance several aspects of bone health in subjects with osteoporosis by acting on different steps in the recruitment and activation of osteoclast cells, increasing mineral density and mechanical strength. Mechanisms that have been suggested involve the downregulation of NF-κB, RANKL, NO production of reactive oxygen species and inflammatory cytokine synthesis (24, 25).

Moreover, a lot of research has recently focused on the use of plum extract due to its high polyphenol content. Graef et al. (26) demonstrated that the polyphenolic extract from dried plums is responsible for reducing osteoclast differentiation and activity through a process of downregulation of osteoclast differentiation acting on the primary bone marrow–cells. In according with other studies, this which was mediated through the suppression of Nfatc1, that is the regulator of osteoclast differentiation process (27).

## Clinical Aspects

### Osteoporosis

Strategies to avoid osteoporosis include decreasing bone loss induced by acute post-menopause estrogen deficiency. As for other chronic diseases, there is increasing indication that inflammation could be part of the etiology of osteoporosis (28). In particular, oxidative stress regulates the promotion of an enhancement in bone resorption, differentiation, and activity of osteoclast cells, so it has a substantial influence on the incidence of osteoporosis. In general, oxidative stress is described as the state of asymmetry between pro-oxidants and antioxidants. It is assumed that there is a correlation between reactive oxygen species (ROS) and its pathogenesis (29). Polyphenols are able of scavenging ROS and downregulating inflammatory cytokine, including osteoclast differentiation factors, including OPG, TNF-α, RANKL (30). For these reasons, antioxidant-rich foods may represent a possible approach for slowing down age-related bone mass reduction and enhancing bone remodeling. Dietary polyphenols have been related with bone health, which may be in part imputable to their antioxidant capability. For this reason, complementary and alternative medicine has generated interest as a natural chance linked to disease prevention. Natural antioxidant supplementation has been investigated to support the reduction of bone loss caused by oxidative stress (31).

**Figure 2:**
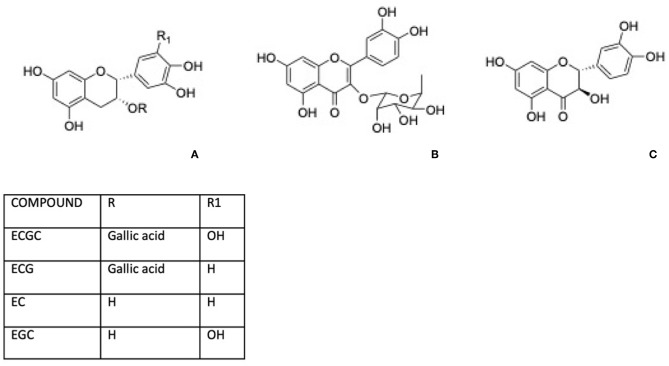
**(A)** Structures of main green tea polyphenols. **(B)** Quercitrin. **(C)** Taxifolin.

Green tea, for instance, is well-known for its content in phenolic compounds including (–)-epigallocatechin 3-gallate (EGCG), (–)-epicatechin gallate (ECG), (–)-epicatechin (EC), and (–)-epigallocatechin (Figure 2A). Shen et al. (32, 33) reported that green tea polyphenols inlet reduces degeneration of bone microarchitecture in rats with chronic inflammation by the downregulation of TNF-α modulating cancellous and endocortical bone compartments. Between Green tea active components, epigallocatechin gallate has attracted attention for its potential health benefits and this molecule was largely investigated for its effect on osteoporosis. The bone formation was affected by EGCG through the enhancement of the alkaline phosphatase activity in osteoblastic cells and bone mineralization, related to the suppression of the osteoclast cells differentiation and of the formation of oxidative stress-induced calcium stone formation in rats (34).

Vester et al. (35) demonstrated that stimulation of primary human osteoblasts with low doses of green tea extracts during oxidative stress over 21 days improved mineralization and had beneficial effect on extra-cellular matrix production with higher gene expression of osteocalcin and collagen1α1 during osteoblasts differentiation.

**Figure 3:**
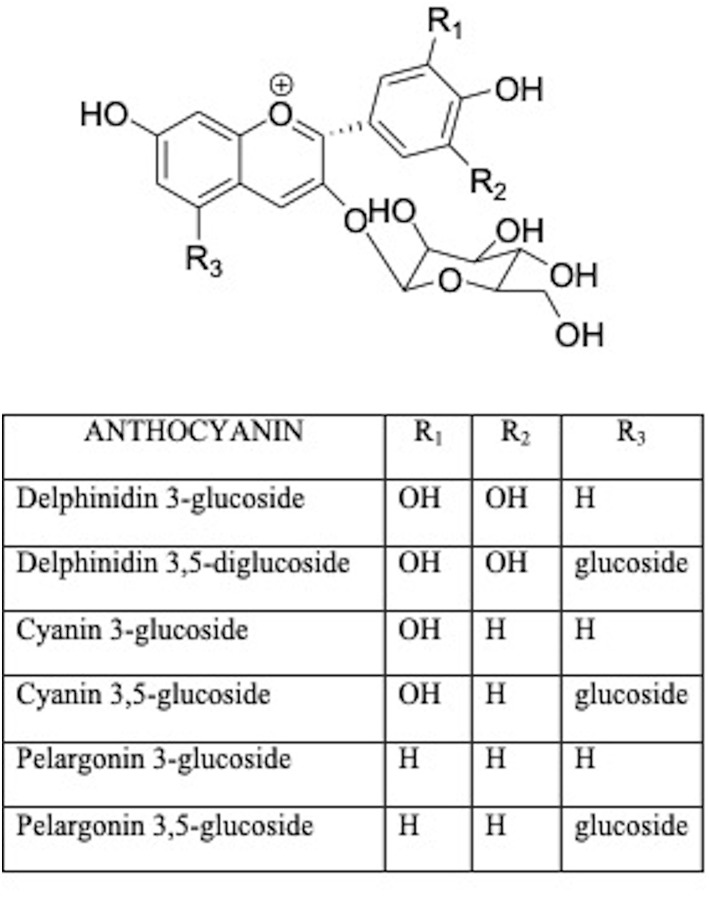
Structures of main anthocyanins.

Anthocyanins are water-soluble glycosides including a variety of compounds comprising pelargonidin, cyanidin, delphinidin, peonidin, petunidin, malvidin (Figure 3) (36). The health effects of anthocyanins are mainly due to their ability to impair oxidative stress (37). Anthocyanin chalcones and quinoidal bases with a double bond linked to the keto group are antioxidants in scavenging free radicals (38). Therefore, berry food and drinks could diminish the effects of age-related bone loss and decrease the risk of osteoporosis in humans (39).

In other studies, Devareddy et al. reported that 5% blueberry treatment (w/w) for 100 days inhibited the loss of whole-body bone mineral density due to the ovariectomy in rats, and suppressed femoral mRNA levels of bone turnover biomarkers that were enhanced by estrogen deficiency (40).

**Figure 4:**
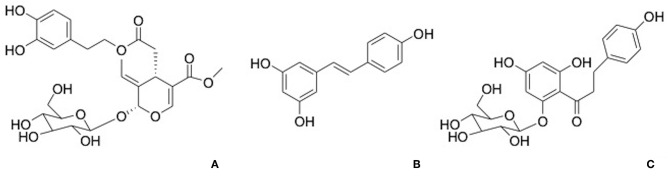
Structures of phloridzin **(A)**, oleuropien **(B)**, and resveratrol **(C)**.

Phloridzin, a dihydrochalcone contained in apples, apples juice, and purees, has been demonstrated to contribute to the antioxidant activity (Figure 4A). The activity of phloridzin on glucose uptake and diabetes has been explored and reported in many research works (41). The phlorizdin intake displayed a protection against ovariectomy-induced osteopenia under inflammatory patterns by the downregulation of inflammation markers and bone resorption (42).

In a murine model, Kim et al. found that phloretin (not glucoside form of phloridzin) inhibited receptor activator of NF-κB ligand (RANKL)-induced formation of multinucleated osteoclasts and diminished bone resorption area produced during the osteoclast differentiation process (43).

Oleuropein (Figure 4B), a secoiridoids esterified with hydroxytyrosol found in the olive tree and derivates, is well-known for its pharmacological activities including antioxidant, anti-inflammatory, anti-cancer, antimicrobial, and antiviral, cardioprotective against acute adriamycin cardiotoxicity, anti-ischemic, and hypolipidemic (44, 45). Puel et al. displayed the dose-dependent bone-sparing effect of oleuropein showing a reduction of bone loss and at the same time a downregulation of inflammatory biomarkers in ovariectomized rats (46).

Resveratrol (Figure 4C) is a stilbene phytoalexin found in many plant species and it is crucial polyphenol found in red wine. It has been considered that dietary resveratrol could affect as an antioxidant, promoting nitric oxide production, platelet aggregation and enhancing high-density lipoprotein cholesterol. In addition, resveratrol was shown to be a chemo preventive agent and exhibits anti-inflammatory, neuroprotective, and antiviral properties (28, 47).

The PGF2α-effect on OPG synthesis was suppresses by resveratrol through the inhibition of the MAP kinase pathways in osteoblasts (48).

Also, resveratrol downregulates bone morphogenic protein-4 (BMP-4) and stimulates VEGF synthesis through the inhibition of p70 S6 kinase in osteoblasts (49) via sirtuin-1 (SIRT1) activation (50).

The effect of above described compounds on osteoblast and osteoclast activity, are summarized in Table 1 (16, 19, 20, 26, 29, 35, 37, 39, 43–45, 50–52).

**Table 1:** Effect of different polyphenols on osteoclasts and osteoblasts activity *in vitro*.

**Compound**	**Effect on osteoclasts**	**Effect on osteoblasts**
Iicarin	− (20)	+ (19)
Green tea polyphenols	− (16)	+ (35)
Anthocyanins	− (29)	+ (37)
Phloridzin	− (43)	+ (39)
Oleuropein	− (45)	+ (44)
Resveratrol	− (51)	+ (50)
Dried plum	− (26)	+ (52)

*–, Reduced cellular activity*.

*+, Enhanced cellular activity*.

### Periodontal Disease

Periodontitis is a devastating inflammatory disease of tooth-supporting tissues, which are composed by cementum, periodontal ligament, and alveolar bone, due to imbalance between oxidative stress and antioxidant activity.

Of note, inflammatory stimulation by periodontal bacteria increases the production of crevicular fluid and modulate the production of leukocytes, which, in order to deactivate periodontal pathogens, liberate single oxygen, and hypochlorous acid into the crevicular fluid (53).

The subsequent oxidative stress is counteracted by the antioxidant activity of ascorbate, albumin, and urate characteristic of the crevicular fluid and derived from plasma. When there is instability between oxidative stress and antioxidant activity, periodontal tissue demolition may appear. These remarks suggest that antioxidant rich diets might protect periodontal tissue from development and progression of pathologies, particularly in subjects exposed to environmental and dietary sources of oxidative stress (54, 55).

Based on clinical studies about the biochemical properties delivery of polyphenols formulations there is emerging line of natural therapies for periodontitis that may maximize and improved oral health among more populations. Actually, administration of tea polyphenols, by holding green or black tea, in the mouth for 2–5 min enhance the antioxidant capacity of saliva, and daily use of two fresh grapefruits for 2 weeks increases the phagocytic capability of the polymorphonuclear leucocytes inside the gingival crevicular fluid (56).

**Figure 5:**
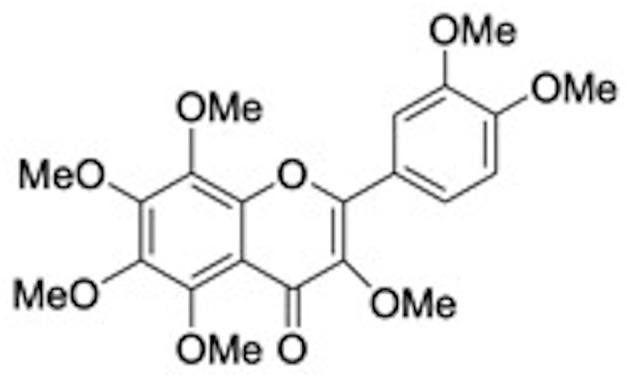
Structure of 3,5,6,7,8,3,4-Heptamethoxyflavone.

Following these suggestions polyphenoids may be employed in dentistry as a prophylaxis against bacterial infection and plaque formation, and as adjuvant therapies to aid post-operative healing of dental sockets and other traumatized tissues. Remarkable, in a recent study researchers find out that quercitrin, a glicoside of quercetin, and ramnose (Figure 2B) present in tartary buckwheat and in the bark of several oak, was found bioactive *in vitro* on human gingival fibroblasts, downregulating the gene expression of markers linked to inflammation and overexpressing genes that modulate different categories of collagen (57). Matsumoto et al. have reported that a citrus flavonoid 3,5,6,7,8,3′,4′-Heptamethoxyflavone (HMF) (Figure 5) found in Valencia oranges clearly suppressed the osteoclast formation and PGE2 production induced by IL-1. In mouse calvarial organ cultures, HMF attenuated the bone resorption elicited by LPS. HMF inhibits bone resorption induced by inflammation preserving bone mass and contributing to keep away from tooth loss (58).

## Potential Applications in Oral Medical Devices

Due to recent progression in molecular biology and in the bone remodeling pathways many polyphenols are believed promising molecules able to act on osteoblast differentiation and on mineralization without the high financial impact of other osteoinductive factors.

These results are correlated with an exciting application of flavonoids like bioactive surfaces that could be an option to the use of growth factors i.e., in oral medical devices (59). Noteworthy, the use of growth factors, such as BMPs, in biomedical devices has lately gained several negative points regarding its stability, administration, bioactivity, and bioavailability. They are frequently very expensive and very limited in respect on the regulatory approval (60). By another side, flavonoid compounds are cheap, bioavailable, easy to find in daily food and, for this reason, probably easily be translated into clinical applications. For this rationale plant-derived products represent an innovative and interesting candidates for biomaterial applications, including dental research fields. Biomolecules can be believed as promising field relating to the improving of the bioactivity of biomaterial, and a safe substitute to pharmaceuticals, animal-derived compounds or growth factors. With the aim to attempt to obtain a faster osteointegration to speed up the overall treatment process, the use of biomimetic agents represents an interesting research area related with implant dentistry. For example, the functionalization with flavonoids conferred an osteopromotive characteristic to the Titanium surface as discussed by Cordoba et al. (61). In their study, researchers created a bioactive interface based on the covalent immobilization of flavonoids taxifolin (Figure 2C) and quercitrin on titanium surfaces.

## Polyphenols Safety and Toxicity

The consumption of food polyphenols such as flavonoids has been associated with a wide range of health benefits both disease preventive and therapeutic agents, including optimizing cardiometabolic health, cancer prevention, and to a lesser extent positively impacting brain functioning in humans.

Diet-derived polyphenols are considered safe based on their long history of use as food or as traditional medicines, recently it is becoming the idea that these specialized metabolites could have toxic effects at pharmacological concentrations, and in several diseases or polypharmaceutical contexts (62).

The risk of toxic effects is increased using pharmacological doses in prevention/treatment and supplement situations and genetic polymorphisms or molecule–drug interactions that decrease/increase the bioavailability. Few reports on toxicity of these compounds are reported, therefore investigations of the side effects of polyphenols is necessary.

## Conclusions

Bone pathologies majorly including osteoporosis osteoarthritis and oral diseases are becoming frequent with the growth in the aging population, a fact which is frightening if we consider the estimations that by 2030 20% of Europeans and 30% of the US population will be over the age of 65. There are only few standard therapies available for the treatment and prevention of these pathologies so a possible protective effect by natural molecules in multifactorial dysmetabolic disease could be a substitute to rise above side effects of conventional therapies.

Majority of the clinical researches underlined in this review, would attempt to demonstrate the relationship between polyphenols intake and bone turnover regulation. However, in spite of abundant *in vitro* and *in vivo* animal models, up to the present time, there is an absence of consistent human studies relating with polyphenol consumption. Although it was largely described the main role of antioxidative mechanism of polyphenols in prevention and treatment of bone remodeling diseases, it would be required more studies focused on the application of these compounds as therapeutic alternative in bone resorption diseases.

## Author Contributions

VN selected studies and wrote the manuscript and takes responsibility for the manuscript. ND, SN, FC, FB, and RD analyzed and compared literature data, and edited the manuscript. All authors approved the manuscript.

### Conflict of Interest Statement

The authors declare that the research was conducted in the absence of any commercial or financial relationships that could be construed as a potential conflict of interest.
